# Molecular Subtypes and CD4^+^ Memory T Cell-Based Signature Associated With Clinical Outcomes in Gastric Cancer

**DOI:** 10.3389/fonc.2020.626912

**Published:** 2021-03-17

**Authors:** Zhi-Kun Ning, Ce-Gui Hu, Chao Huang, Jiang Liu, Tai-Cheng Zhou, Zhen Zong

**Affiliations:** ^1^ Department of Gastrointestinal Surgery, The Second Affiliated Hospital of Nanchang University, Nanchang, China; ^2^ Department of Day Ward, The First Affiliated Hospital of Nanchang University, Nanchang, China; ^3^ Department of Gastroenterological Surgery and Hernia Center, The Sixth Affiliated Hospital of Sun Yat-sen University, Guangdong institute of Gastroenterology, Guangdong Provincial Key Laboratory of Colorectal and Pelvic Floor Diseases, Guangzhou, China

**Keywords:** gastric cancer, prognostic signature, CD4^+^ memory T cell, tumor microenvironment, weighted gene co-expression network analysis

## Abstract

**Background:**

CD4^+^ memory T cells are an important component of the tumor microenvironment (TME) and affect tumor occurrence and progression. Nevertheless, there has been no systematic analysis of the effect of CD4^+^ memory T cells in gastric cancer (GC).

**Methods:**

Three datasets obtained from microarray and the corresponding clinical data of GC patients were retrieved and downloaded from the Gene Expression Omnibus (GEO) database. We uploaded the normalize gene expression data with standard annotation to the CIBERSORT web portal for evaluating the proportion of immune cells in the GC samples. The WGCNA was performed to identify the modules the CD4^+^ memory T cell related module (CD4^+^ MTRM) which was most significantly associated with CD4^+^ memory T cell. Univariate Cox analysis was used to screen prognostic CD4^+^ memory T cell-related genes (CD4^+^ MTRGs) in CD4^+^ MTRM. LASSO analysis and multivariate Cox analysis were then performed to construct a prognostic gene signature whose effect was evaluated by Kaplan-Meier curves and receiver operating characteristic (ROC), Harrell’s concordance index (C-index), and decision curve analyses (DCA). A prognostic nomogram was finally established based on the CD4^+^ MTRGs.

**Result:**

We observed that a high abundance of CD4^+^ memory T cells was associated with better survival in GC patients. CD4^+^ MTRM was used to stratify GC patients into three clusters by unsupervised clustering analysis and ten CD4^+^ MTRGs were identified. Overall survival, five immune checkpoint genes and 17 types of immunocytes were observed to be significantly different among the three clusters. A ten-CD4^+^ MTRG signature was constructed to predict GC patient prognosis. The ten-CD4^+^ MTRG signature could divide GC patients into high- and low-risk groups with distinct OS rates. Multivariate Cox analysis suggested that the ten-CD4^+^ MTRG signature was an independent risk factor in GC. A nomogram incorporating this signature and clinical variables was established, and the C-index was 0.73 (95% CI: 0.697–0.763). Calibration curves and DCA presented high credibility for the OS nomogram.

**Conclusion:**

We identified three molecule subtypes, ten CD4^+^ MTRGs, and generated a prognostic nomogram that reliably predicts OS in GC. These findings have implications for precise prognosis prediction and individualized targeted therapy.

## Introduction

Gastric cancer (GC) is the fifth most diagnosed malignancy and is the third highest cause of cancer mortality worldwide (1). The incidence of GC greatly varies among regions, with more than 70% of cases occurring in developing countries, mainly in Eastern Asia (2). The prognosis of GC patients is still not optimistic owing to genetic heterogeneity and the difficulty of early-stage screening, especially in China (3). Therefore, the identification of effective biomarkers is of great importance to better evaluate tumor progression, predict overall survival and enhance therapeutic efficacy.

After years of in-depth research, the scientific understanding of tumor progression has become more comprehensive and recognizes single malignant cells and the very complex niche called the tumor microenvironment (TME). The TME has a considerable impact on the occurrence and development of GC (4). Disorders of the immune system can enable tumor cells to evade immune surveillance. Molecular profiles of immune cells and immune-related genes (IRGs) within their TME represent promising candidates for predictive and prognostic biomarkers (5, 6). Recently, T cell immunity has been an area of active research. T cells progressively lose function and become exhausted during cancer; however, effective T cell responses are essential to ultimately controlling tumors (7). Several studies have revealed a relationship between T cell immunity and tumor development. This has been found in lung cancer (8), breast cancer (9), and ovarian cancer (10). CD4^+^ memory T cell has been reported to be an important role in TME. In colorectal cancer, it has been suggested in more infiltrated than normal tissue (11); in triple-negative breast cancer, CD4^+^ memory T cell enrichment score seem higher in invasive tumors (12); in lung adenocarcinoma, it seemed relative hypometabolism and favorable prognosis (13), but the relationship is not yet clear in the case of GC. Subsequently, through the bioinformatics tool of CIBERSORT and Kaplan-Meier survival curves, we explored the relationship between immune infiltration and outcome of patients with gastric cancer according to the gene expression profiles from the GEO database and found that prognosis were closely associated to memory CD4^+^ T cells.

Collectively, in this study, we investigated the effects of CD4^+^ memory T cells in GC patients. We explored the potential role of CD4^+^ memory T cells, CD4^+^ memory T cell-related genes, and molecular subtypes in GC using bioinformatics models. The results will contribute to the development of precision therapy strategies for gastric cancer patients.

## Materials and Methods

### Datasets and Patients

The public microarray data sets and corresponding clinical data of GC patients were retrieved and downloaded from the Gene Expression Omnibus (GEO) database (https://www.ncbi.nlm.nih.gov/geo/). Clinical data contained the age, gender, pathologic TNM stage, and survival information. Three datasets were selected to merge into a single cohort for further analysis: GSE34942, GSE57303, and GSE62254, for a total of 426 gastric cancer samples. Another three datasets (GSE26899, GSE84437, and GSE26901) and the Cancer Genome Atlas (TCGA) transcriptome data which was downloaded from https://portal.gdc.cancer.gov/repository, were performed as external validation. Series matrix files and data tables of the microarray platform were downloaded from the GEO website. The preprocessing of data was as follows: (1) GC samples without clinical survival information were removed; (2) data on normal GC tissue samples were removed; (3) and only the expression profile of immune-related genes was included.

### Estimation of Immune Cell Type Fractions

Batch effects and noise were inherent in the three datasets (GSE34942, GSE57303, and GSE62254), which were from multiples studies spanning diverse cell lines and different platforms; therefore, combat normalization in the “sva” R package was used to co-normalize the three datasets into a single cohort. To quantify the proportion of immune cells in the GC samples, we uploaded the normalize gene expression data with standard annotation to the CIBERSORT web portal (https://cibersort.stanford.edu/), and the algorithm was run using 1000 permutations and the LM22 gene signature as previously described (14). Only samples that had a CIBERSORT output of P < 0.05 were considered in the subsequent Kaplan-Meier (K-M) analysis (**Supplementary Figure 2**).

### WGCNA Network Analysis

The WGCNA R package was used to build a gene co-expression network to mine their module membership associated with immune cells. Immune-related gene (IRG) data were obtained from the InnateDB (https://www.innatedb.ca/). The soft threshold parameter was first applied to ensure a scale-free network. To further identify the functional blocks in the co-expression network of the immune-related genes, the topological overlap measure (TOM) was then performed to calculate the correlation between genes. Finally, connecting modules with the immune cells identified the key module that was most associated with OS.

### Bioinformatics Analysis

After the key module had been identified, the genes were input to Metascape (https://metascape.org/) for gene annotation and gene list enrichment analysis (15), which included biological process (BP), cellular component (CC), molecular function (MF), and Kyoto Encyclopedia of Genes and Genomes (KEGG) pathway analysis. The top 20 terms were selected for visualization; however, more than 20 GO terms or pathway annotations were identified. Unsupervised clustering analysis *via* the “ConsensusClusterPlus” R package was used to perform consensus molecular subtyping of immune subtypes (16). Differentially expressed gens (DEGs) analysis of the three clusters which took the intersection after comparing the two groups separately including intersection of up-regulated IRGs and intersection of down-regulated IRGs, was carried out using the “limma” R package, according to the thresholds of |log2-fold-change| > 0.5 and an adjusted false discovery rate (FDR) P-value of < 0.01. The differentially expressed IRGs (DEIRGs) were obtained by intersecting the list of previously acquired immune-related genes with the list of DEGs. The “clusterProfiler” R package was used to perform Gene Ontology function enrichment, which has been descried in detail in a previous study (17, 18).

### Construction and Validation of Immunoscore Prognostic Model

Univariate Cox regression analysis was used to calculate the hazard proportions for genes of the yellow module; these were considered statistically significant at p<0.05. Least absolute shrinkage and selection operator (LASSO) regression analysis was performed with the “glmnet” R package (19) to select the most useful gens with the best predictive performance using 10-fold cross validation. An immunoscore model of GC patients was then established based on linearly combining the multiplication of the Cox coefficient (β) derived from the LASSO regression analysis by its scale expression value, i.e., immunoscore = Σ Cox coefficient of gene Xi × scale expression value of gene Xi. Patients were classified into high- or low-risk groups according to the optimal cutoff value. The cutoff value was determined based on the association with overall survival (OS) using the “survminer” R package. Time independent receiver operating characteristic (ROC) curves were used to depict the sensitivity and specificity of the survival prediction based on the immunoscore, with quantification of the area under the curve (AUC) using the “timeROC” R package.

### Construction and Validation of Nomogram Model

Multivariate Cox regression analysis was employed *via* the “rms” R package to determine independent prognostic factors, resulting in an immunoscore-based prognostic nomogram with three factors (p < 0.05); only patients with complete clinical data were included. The predictive accuracy of the nomogram was measured and compared through the AUC of the ROC curve, Harrell’s concordance index (C-index), and decision curve analysis (DCA).

## Results

### Composition of Immune Cells in GC

**Figure 1:**
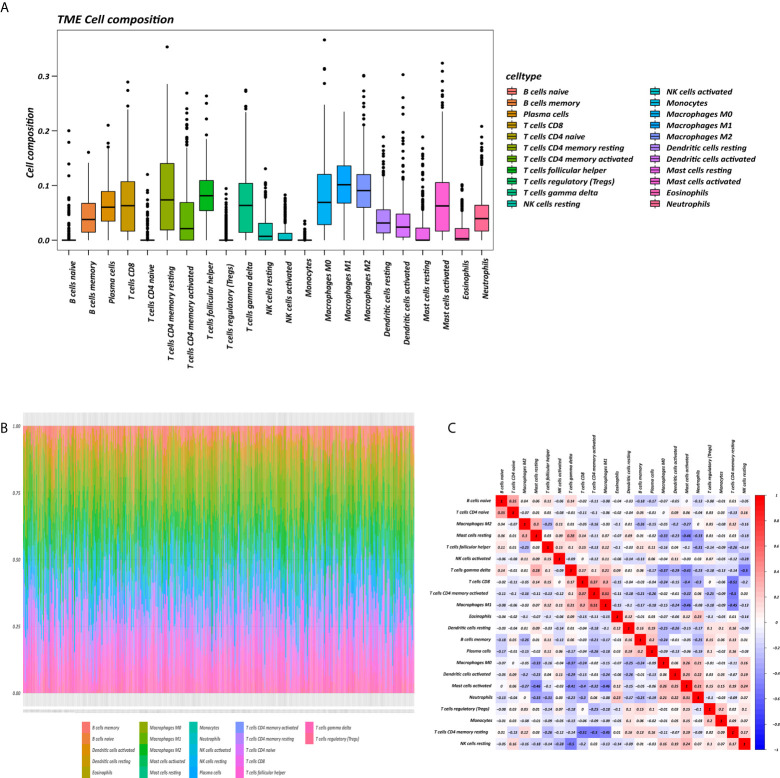
Tumor microenvironment (TME) composition of GC. **(A)** Box plot of immune cell composition. **(B)** Relative proportion of immune cells in each sample. **(C)** Correlation matrix of immune cells.

The proportions of 22 immune cell types in GC assessed by the CIBERSORT algorithm are displayed in a box plot (**Figure 1A**) and heatmap (**Figure 1B**). The 22 tumor immune cell types were weakly or moderately correlated with each other (**Figure 1C**).

### Identification and Functional Enrichment Analysis of CD4^+^ MTRM

**Figure 2:**
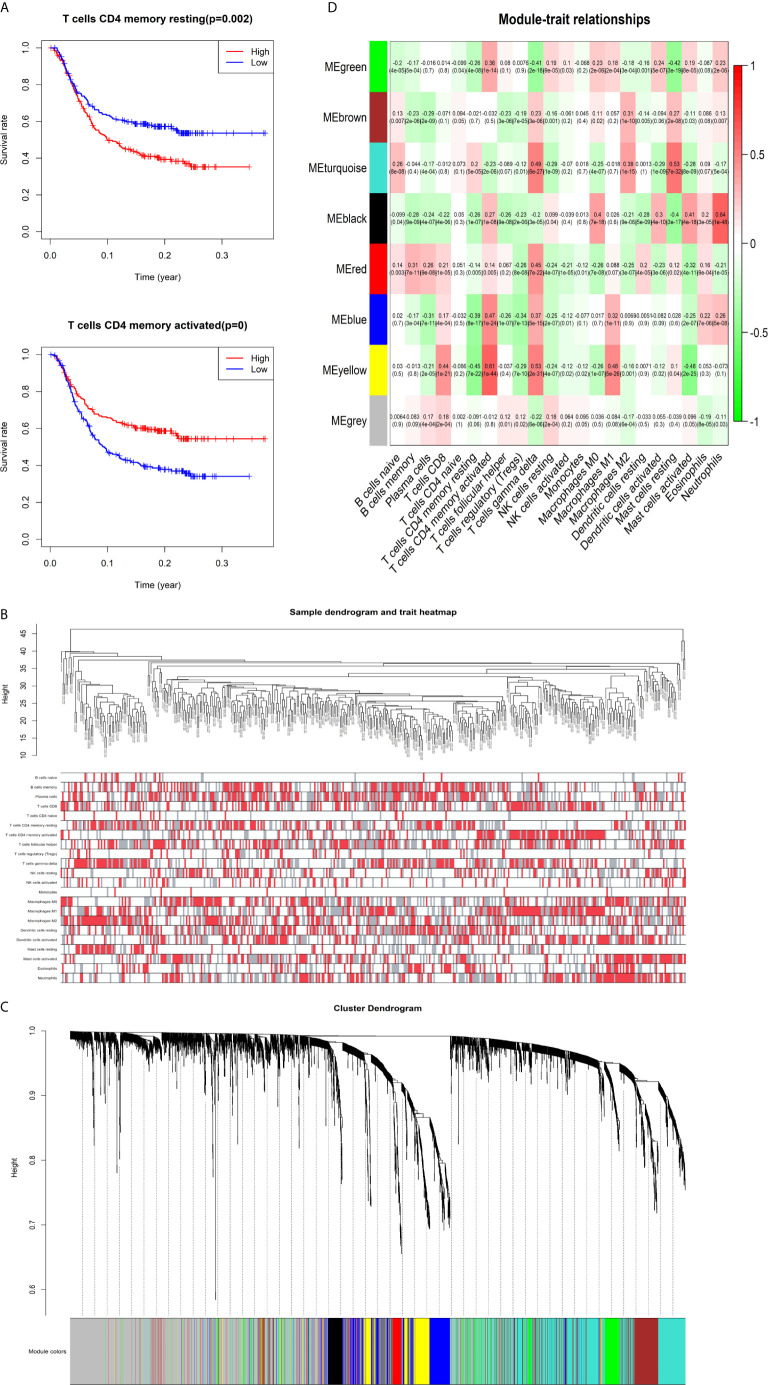
Identification of key gene module associated with overall survival (OS). **(A)** Kaplan-Meier survival curve of two immune cells: CD4 memory resting T cells, CD4 memory activated T cells, and M1 macrophages. **(B)** Sample clustering and corresponding external traits. **(C)** Average linkage hierarchical clustering dendrogram of the genes. **(D)** Heatmap of the correlation between module eigengenes and immunocytes.

**Figure 3:**
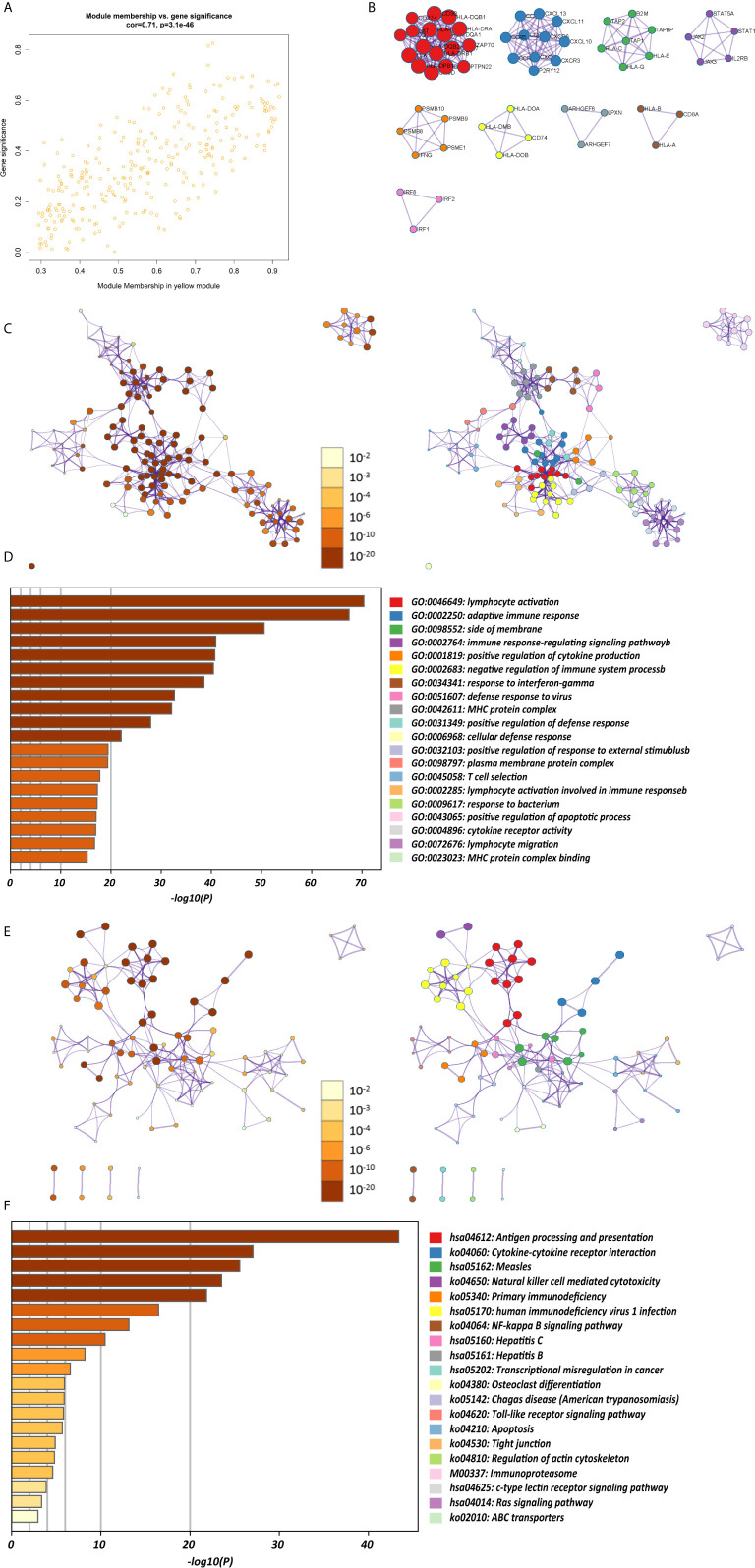
Functional and pathway enrichment analysis in the CD4^+^ MTRM. **(A)** Scatter plot of gene significance versus module membership in the CD4^+^ MTRM. **(B)** Subnetwork of gene in the CD4^+^ MTRM. **(D, F)** GO and KEGG enrichment analysis for genes in the CD4^+^ MTRM. **(C, E)** Interaction network of enriched biological processes in the CD4^+^ MTRM.

The K-M survival curves with log-rank tests for the 22 immune cell types are presented on **Supplementary Figure 2**. These results indicated that high CD4 memory resting T cells, low CD4 memory activated T cells and low M1 macrophages were significantly associated with poorer OS, p = 0.002, p < 0.001, p = 0.029, respectively (**Figure 2A**). WGCNA was then performed to select modules of highly correlated genes related to external sample characteristics (prognostic immunocytes). After placing the IRGs with similar expression patterns into modules by average linkage clustering and determining the soft threshold parameter, we finally identified 8 modules; each colour represents a different module (**Figures 2B, C**). In addition, the relationships between genes and immunocytes in each modules were identified and are displayed in a heatmap (**Figure 2D**). The yellow module is identified as the CD4^+^ memory T cell-related module (CD4^+^ MTRM). This module was most negatively associated with resting CD4^+^ memory T cells, while it was most positively associated with activated CD4^+^ memory T cells and M_1_ macrophages, as assessed by the heatmap and scatter plot scores (**Figure 3A**). Interaction network of gene in the CD4^+^ MTRM were shown in **Figure 3B**. These results suggested that the genes in CD4^+^ MTRM were closely related with OS; therefore, CD4^+^ MTRM was chosen for further analysis. We loaded the genes in the CD4^+^ MTRM into Metascape to explore the underlying biological processes. The Metascape results revealed functional categories mainly related to immune regulation, such as the negative regulation of the immune system process and antigen processing and presentation (**Figures 3D, F**). Moreover, the enriched processes were highly associated and clustered into an intact network (**Figures 3C, E**).

### CD4^+^ MTRM-Based Clusters Were Significantly Associated With Prognosis, Clinicopathological Characteristics, and Immunocytes

**Figure 4:**
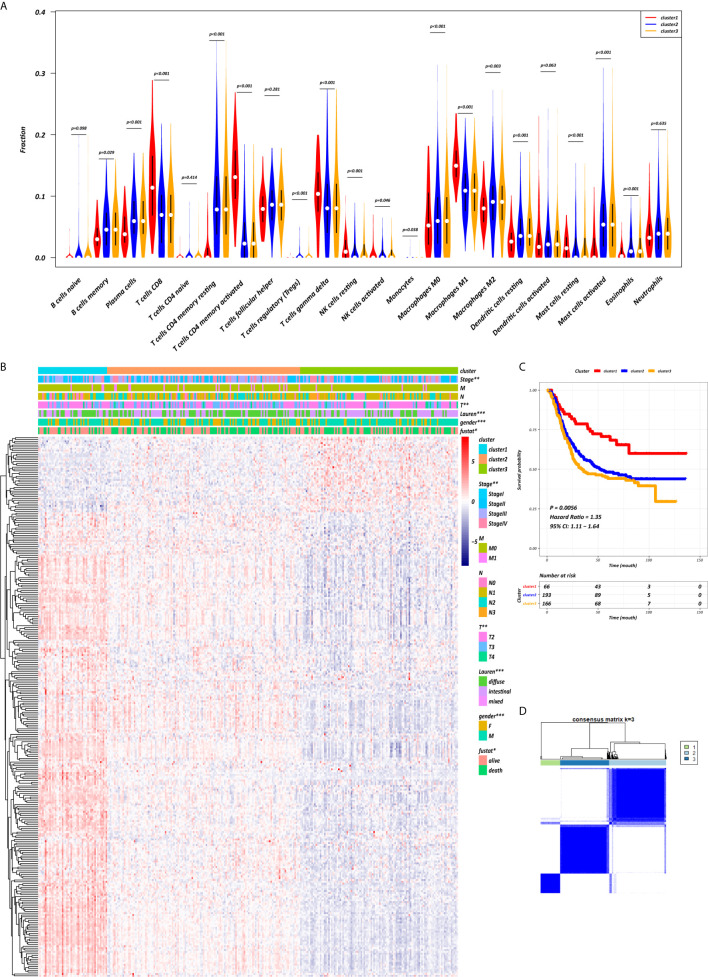
CD4^+^ MTRM-based clusters significantly associated with prognosis, clinicopathological characteristics and immunocytes. **(A)** Violin plot comparing immunocyte proportions between the three clusters. **(B)** Unsupervised hierarchical clustering of CD4^+^ MTRM gene subsets in patients with gastric cancer. **(C)** Kaplan-Meier analysis with log-rank test of the three clusters. **(D)** Consensus matrixes of GC samples in CD4^+^ MTRM.

The abundance of tumor-infiltrating immunocytes varied considerably by individual. To gain greater insight into the molecular heterogeneity of GC, we performed unsupervised consensus analyses with the k-means algorithm of the yellow module patient samples based on the immunocyte proportion (**Figure 4B**). Three distinct molecular clusters were identified (k-means = 3, **Figure 4D**). The cell proportions of each immune subtype are shown in **Figure 4A**. To further elucidate the clinical significance of the identified clusters, we explored the correlation between the cluster and clinicopathological features. The clusters were related with distinct patterns of survival in the Kaplan-Meier analysis (**Figure 4C**). For example, cluster 1, defined by high levels of activated CD4^+^ memory T cells and M_1_ macrophages and low levels of CD4^+^ memory resting T cells (all p < 0.001), was significantly associated with a better prognosis compared with the other clusters (p = 0.0056).

**Figure 5:**
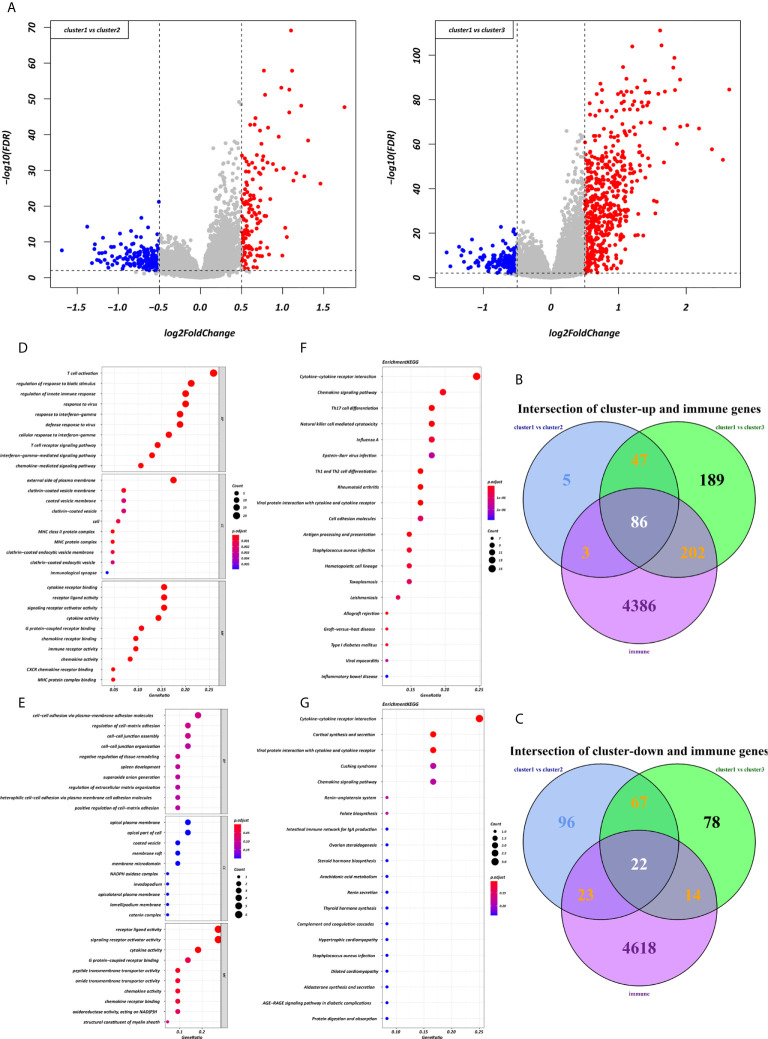
Expression and enrichment analyses of DEIRGs between the three clusters. **(A)** Volcano plot of DEGs. **(B, C)** Venn diagram of up- and downregulated DEIRGs. **(D, E)** Dot plot showing the top 10 most significant GO terms of up- and downregulated DEIRGs, including BP, CC, and MF. **(F, G)** The top 20 KEGG pathways of up- and downregulated DEIRGs that are shown in the dot plot.

Using the “limma” R package, we screened 222 DEGs among the three clusters by taking the intersection after comparing the two groups separately, including 133 up-regulated genes and 89 down-regulated genes that met the thresholds of |log2 FC| > 0.5 and adjusted P < 0.01 (**Figure 5A**). Upon further comparison with the list of IRGs, 108 DEIRGs were obtained; 86 were upregulated and 22 were downregulated (**Figures 5B, C**). To understand how these DEIRGs might drive GC development, functional enrichment analyses were performed. The results revealed that upregulated DEIRGs were mainly involved in T cell activation, regulation of responses to biotic stimuli, and regulation of the innate immune response. Downregulated DEIRGs were closely associated with receptor ligand activity, signalling receptor activator activity, and cytokine activity based on the top three terms confirmed in the GO analyses (**Figures 5D, E**). According to the KEGG pathway analysis results, we identified most significantly enriched pathways (**Figures 5F, G**). The cytokine-cytokine receptor interaction was the most enriched pathway in both the up- and downregulated DEIRGs. A difference of P < 0.05 indicated statistical significance. Moreover, the expression levels of seven immune checkpoint genes (PD-L1, CTLA4, HAVCR2, IDO1, PD1, PD-L2, and TIGIT) were analyzed in the 3 clusters (**Supplementary Figure 3A**). Cluster 1 showed more enrichment in immune checkpoint genes (all p < 0.05) and therefore might be more responsive to immunotherapy.

### Construction and Validation of Immunoscore Prognostic Model

**Figure 6:**
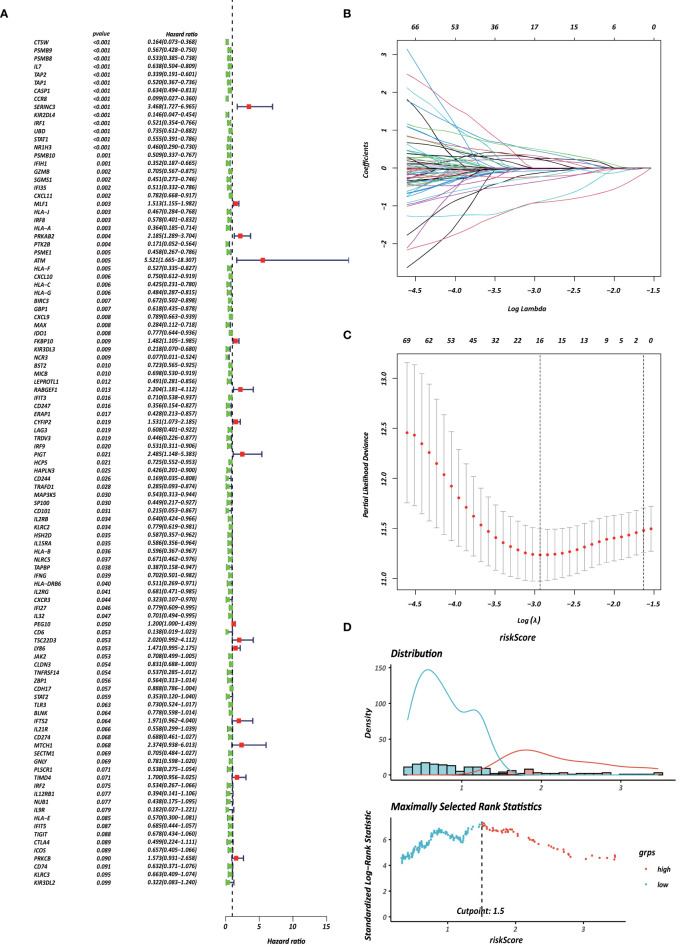
Identification of survival-associated genes in CD4^+^ MTRM. **(A)** Forest plot of prognosis-related genes with univariate Cox regression analysis. **(B, C)** LASSO analysis result. **(D)** Immune risk score distribution of 10 CD4^+^ MTRG signature.

**Figure 7:**
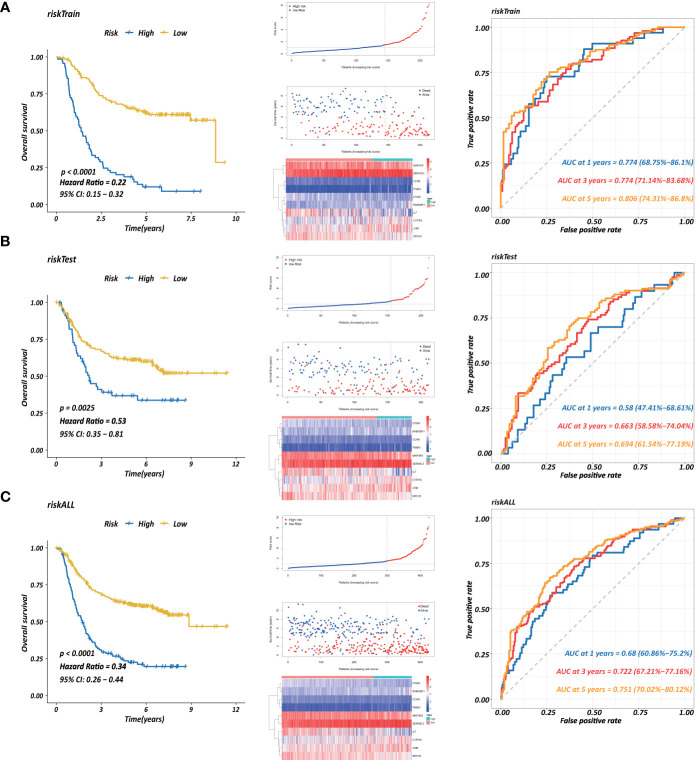
Establishment of a prognostic model based on CD4^+^ MTRGs in GC, including Kaplan-Meier analysis between high-risk and low-risk groups of patients with GC; risk score distribution of patients in high- versus low-risk group; the scatter plots for survival status in GC high-risk group and low-risk group; the heatmaps of CD4^+^ MTRG expression between the high-risk and low-risk groups; time-dependent ROC curves of the OS signatures at 1, 3, and 5 years. Ten CD4^+^ TRG prognostic model constructed using training set **(A)**, testing set **(B)**, and the entire sample set **(C)**.

Each gene in the CD4^+^ MTRM was analyzed by univariate Cox regression analysis. We identified 105 genes that were significantly associated with the prognosis of gastric cancer (**Figure 6A** and **Supplementary Table 1**) that were defined as CD4^+^ memory cell-related genes (CD4^+^ MTRGs). In the subsequent LASSO regression analysis and multivariate Cox analysis (**Figures 6B, C**), 10 CD4^+^ MTRGs were identified (**Table 1**), which indicated that these CD4^+^ MTRGs would be selected to establish a prognostic model. The downregulated expression of IL-1, CTSW, NR1H3 and CCR8 (with HR < 1) indicated these molecules as tumor suppressors, whereas the upregulated expression levels of *LY86*, *RABGEF1*, *CYFIP2* and *SERINC3* (with HR > 1) indicated these molecules as oncogenes. The patients were separated into training and validation cohort in a ratio of 1:1 using the stratified randomization method. We constructed a prognostic model in training cohort that divided patients into high- and low-risk groups based on the optimal cutoff value of the immune-risk score that was calculated by the “survminer” R package (**Figure 6D**). The Immune-risk score = 0.87096 × *LY86* expression + (−0.446079131) × *IL7* expression + (−1.182540714) × *CTSW* expression + (−0.690489234) × *NR1H3* expression + (−1.49913792) × *CCR8* expression + 0.728725268 × *RABGEF1* expression + 0.420982806 × *CYFIP2* expression + 0.800957561 × *SERINC3* expression + 0.649569536 × *TIMD4* expression + (−0.565943213) × *MAP3K5* expression. The results showed that high score patients had a worse OS than those of low score patients (p < 0.0001). The area under the ROC curves (AUC) of the prognostic model for OS, assessed as a continuous variable, was investigated in the training cohort by using time‐dependent ROC analysis at the time points 1, 3 and 5 years (AUC: 0.774, 0.6875–0.8610, 95%CI; 0.774, 0.7114–0.8368, 95%CI, and 0.806, 0.7431–0.8680, 95%CI, respectively, **Figure 7A**). To determine if the immune-risk score model is solid in different populations, the same formula was applied to the validation cohort and also to the entire cohort. The patients were then divided into high- or low-risk groups using the cut off value obtained from training cohort. Consistent with the findings in the training cohort, in both the validation cohort and entire cohort the K-M curves illustrated that the high-risk group was associated with a notably poorer prognosis than the low-risk group (all p < 0.001). The results revealed that the predictive potential of the immune-risk score model is applicable in different populations. The prognostic accuracy of the immunoscore in the validation cohort and the entire cohort was also evaluated; the AUC achieved 0.58 (0.4741–0.6861, 95%CI); 0.663 (0.5858–0.7404, 95%CI), and 0.694 (0.6154–0.7719, 95%CI) in the validation cohort and 0.68 (0.6086–0.7520, 95%CI); 0.722 (0.6721–0.7716, 95%CI) and 0.751 (0.7002–0.8012, 95%CI) in the entire cohort at 1, 3, and 5 years, respectively (**Figures 7B, C**).

**Table 1:** The results of multivariate Cox regression analyses.

id	coef	HR	HR.95L	HR.95H	pvalue
LY86	0.87096	2.389203387	1.403461238	4.067296389	0.001333693
IL7	-0.446079131	0.640133115	0.485773326	0.843542416	0.001532239
CTSW	-1.182540714	0.306499022	0.135934623	0.691079641	0.004362266
NR1H3	-0.690489234	0.501330741	0.30266654	0.830394109	0.007322481
CCR8	-1.49913792	0.223322599	0.059804773	0.833929817	0.025739371
RABGEF1	0.728725268	2.072437122	1.041916035	4.122208971	0.037801916
CYFIP2	0.420982806	1.523458084	1.01971892	2.276043416	0.039850263
SERINC3	0.800957561	2.22767304	1.022095648	4.855247338	0.043910504
TIMD4	0.649569536	1.914716435	0.936923802	3.912953241	0.074864124
MAP3K5	-0.565943213	0.567824315	0.300613298	1.072555521	0.081140893

Furthermore, three external datasets GSE26899, GSE84437, and GSE26901 and the TCGA transcriptome data were used to confirm the association between the immunoscore prognostic model and survival outcomes in GC patients. Immune-risk scores were calculated with the same formula for each patient. Patients were divided into high- and low-risk groups according to the optimal cutoffs identified for each dataset. The KM survival curves revealed significant difference in OS between groups in both datasets. High-risk groups had markedly poorer outcomes than low-risk groups (**Supplementary Figure 5**).

All these suggested that the 10 DEIRG signature had high sensitivity and accuracy and could be used for monitoring survival. The expression of five immune checkpoint genes (PD-L1, CTLA4, HAVCR2, IDO1, PD1, PD-L2, and TIGIT) were assessed in the high- and low-risk groups (**Supplementary Figure 3B**). The patients in the low-risk group were more enriched in these immune checkpoint genes (all p < 0.05) and therefore might be more responsive to immunotherapy.

### Relationship Between Immune Infiltration and the Prognostic Signature

To further explore whether CD4^+^ MTRGs reflected the status of GC TME, an association analysis was used to evaluate the relationship between CD4^+^ MTRGs in the prognostic model and immune cell infiltration. The risk factors based on the model were positively associated with naive B cells (r = 0.296, P < 0.001), activated mast cells (r = 0.137, P = 0.005), M2 macrophages (r = 0.234, P < 0.001), naive CD4 T cells (r = 0.097, P < 0.05), and resting memory CD4 T cells (r = 0.136, P = 0.005). They were negatively related with memory B cells (r = −0.110, P = 0.024), M1 macrophages (r = −0.113, P = 0.020), plasma cells (r = −0.169, P < 0.001), CD8 T cells (r = −0.186, P < 0.001), and activated memory CD4 T cells (r = −0.247, P < 0.001) (**Supplementary Figure 4**).

### Construction and Validation of CD4^+^ MTRG-Clinical Nomogram Model

**Figure 8:**
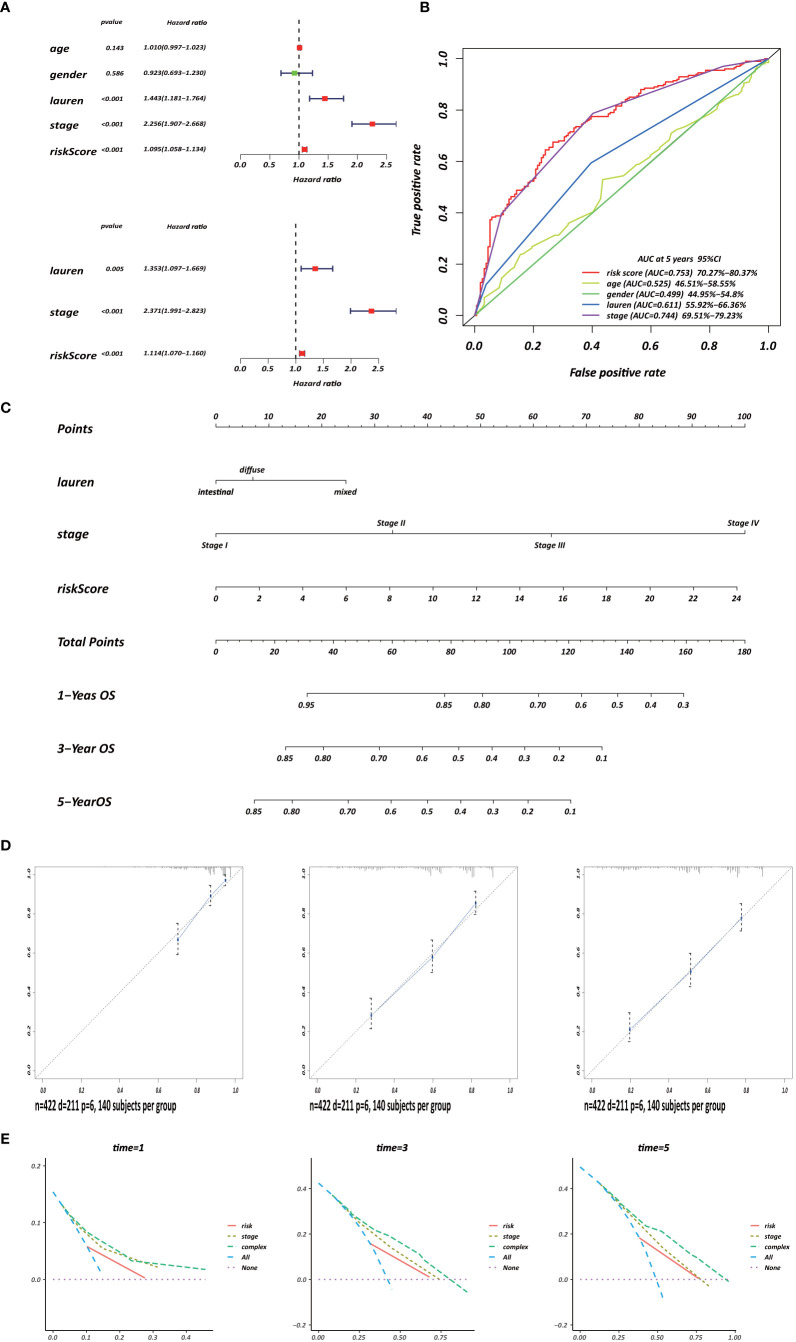
Development of a CD4^+^ MTRG clinical nomogram to predict OS in GC patients. **(A)** Forest plot visualization of prognosis-related factors based on the univariate and multivariate Cox regression analysis. **(B)** ROC curve analysis of the independent prognostic factors. **(C)** Nomogram of GC patient OS combining the risk score and two clinicopathological variables. Calibration curves **(D)** and DCA **(E)** of the nomogram at 1, 3, and 5 years.

Several clinicopathological variables are independent features in GC patient prognosis; these include tumor site, tumor size, and TNM stage (20, 21). Therefore, we used clinicopathological variables and the risk score to construct a nomogram to obtain a more accurate prediction of GC prognosis. First, we used univariate and multivariate Cox regression analysis to identify three independent OS factors: Lauren histologic type, stage, and risks score (**Figure 8A**). A CD4^+^ MTRG-clinical nomogram was then developed based on these independent factors for OS are shown in (**Figure 8C**). AUCs for the nomogram at 5 years were 0.611 (55.92–66.36%, 95%CI), 0.744 (69.51–79.23%, 95%CI), and 0.753 (70.27–80.37%, 95%CI) for Lauren histologic type, stage, and risk score, respectively (**Figure 8B**). The C-index was 0.73 (95% CI: 0.697–0.763) for the OS nomogram. We also established calibration curves and the DCA of the nomogram at 1, 3, and 5 years (**Figures 8D, E**). These results presented high credibility.

## Discussion

Gastric cancer is a prevalent malignant tumor with high recurrence. The prognosis of GC exhibits a wide range, from less than 5 months to over 10 years (1, 22). Precise prognosis prediction and risk stratification can help to determine which patients would benefit from more radical treatment, such as immunotherapy. The efficacy of adjuvant therapy for GC was controversial for many years until Macdonald and Smalley et al. (23, 24). found that postoperative chemotherapy and chemoradiotherapy was a rational standard therapy strategy for GC. Unfortunately, due to tumor heterogeneity, the prognosis can vary widely among patients of the same GC stage who undergo the same adjuvant therapy (25, 26). Therefore, a sensitive and reliable prognostic signature is desired to identify patients who might benefit from adjuvant treatment. Tumor progression develops in the complex tissue microenvironment on which they rely to support their proliferation, infiltration and metastasis (27–29). Different from cancer cells, stromal cell types of TME are stable at the genetic level, which indicates that the TME is a potential therapeutic target (30). Furthermore, accumulating research has confirmed that the TME plays a significant role in predicting patient prognosis (31–33). Owing to this particular insight of the TME, this study selected key genes by screening for gene modules that were significantly correlated with prognostic-related immunocytes. Immune subtypes were then identified and classified, and a risk score model was established on the basis of 10 CD4^+^ MTRGs in the key gene module to evaluate patient prognosis. The efficacy of this prediction strategy was proved in two internal datasets. Nomograms can fit several independent prognostic factors, including molecular and clinicopathological features, which are widely used to assess prognosis in clinical oncology (34). With the ability to calculate an individual numerical probability of a clinical event, nomograms may be more effective than individual prognostic factors for prognosis prediction.

Three immune molecular subtypes were identified in the present study, cluster 1/2/3, by unsupervised clustering analysis based on the expression of the most aberrant immune genes belonging to the yellow module. There were significant differences among the three immune subtypes, including differences in immune status, biological processes and prognosis. Patients in the cluster 1 subtype with the best prognosis suffered from a hyperactivated immunocompetent status. Functional enrichment analyses were applied to the DEIRGs among the three subtypes to elucidate underlying mechanisms. The DEIRGs were found to be significantly associated with T cell activation and cytokine-cytokine receptor interaction, consistent with previous reports. CD8^+^ cytotoxic T cells bind MHC I-presented antigens; these antigens enable the T cells to target tumor cells. Additionally, CD4^+^ T cells have complex and important biological functions in the TME. Clinical research has indicated that combined immunotherapy strategies are effective in treating metastatic cancers because they promote T cell activation (35). Gastric cancer is associated with immune system evasion. Immune checkpoints are inhibitory pathways that can prevent tissue damage by controlling the intensity of the physiological immune response. Especially when the immune system is fighting an infection, these inhibitory pathways are essential for maintaining self-tolerance and physiological homeostasis. In addition, immune checkpoint pathways may also cause immune escape of cancer cells (36). We evaluated the distribution of immune checkpoint gene expression among the three clusters. The subtype with the highest expression of programmed death ligand 1 (PD-L1) provided the best prognosis, and a similar conclusion was obtained in previous research (37, 38). However, others have indicated that programmed cell death protein-1 (PD-1)/PD-L1 expression in cancer cells is significantly associated with poor prognosis (39, 40), and Kawazoe et al (41) found that PD-L1 had no effect on gastric cancer prognosis. To achieve precision therapy, a larger cohort and more controlled research should be applied to clarify the function of PD-L1 in GC.

The heterogeneity of immune cell proportions in different cancers results in a complex immune network in the TME and differentially affects tumor occurrence and progression. Some studies have reported an association between immune cell populations and the prognosis of cancer progression (42–45). CD4 T cells act on tumor immunity by secreting various cytokines or by activating other immune cells (7). CD4^+^ regulatory T cells are the main cells involved in self-tolerance and inhibit tumor immunity (46, 47). Regarding the impact of CD4^+^ T cells in GC patient prognosis, several opposite conclusions have been reported. Shen et al. indicated that CD4^+^ T cells were related with more advanced stages of gastric cancer (48); Kindlund et al. also found that CD4^+^ regulatory T cells can enhance tumor proliferation mediated by IL-10 and TGF-β (49). However, Wang et al. showed that GC patients with higher levels of CD4+ T cells were associated with a good prognosis (50), which was consistent with the findings of the current study.

A novel CD4^+^ MTRG signature that can predict OS in GC patients was constructed through the use of univariate Cox regression analysis and LASSO regression analysis. This CD4^+^ MTRG signature was an independent prognostic factor of GC. Patients with a high immune risk score had significantly poorer outcomes than those in the low-risk group. IL-7, CTSW, NR1H3 and CCR8 were downregulated; these were considered to be protective genes. LY86, RABGEF1, CYFIP2 and SERINC3 were upregulated and were related with a poor outcome. IL-7 has been found to play an anti-tumor role in melanoma (51). By contrast, others have shown that IL-7 might have a pro-tumor function. By limiting p27kip, IL-7 was shown to promote lung cancer proliferation (52) and accelerate bladder cancer invasion and migration (53). Nathan suggested that blocking the CCR8-CCL1 interaction, alone or combined with other immune checkpoint inhibitors, was a therapeutic strategy for malignant diseases (54). However, the role of IL-7 and CCR8 in gastric cancer development has not yet been clarified. Furthermore, there are few studies of the other eight genes in the ten-gene signature. The potential biological function of these genes in GC requires further clarification through experimental research.

A nomogram is a practical and intuitive evaluation approach. Here, we established a nomogram with meaningful AUC values based on the expression levels of genes in a selected panel. The nomogram was used to evaluate the deterioration and outcome of patients in this study, which is more economical and clinically practical than whole-genome sequencing. DCA and calibration curves showed the efficacy of this nomogram. A ten-gene signature and clinicopathological variables were integrated into the graphical scoring system, which was easy to understand. This scoring system could be used to facilitate individual treatment and determine a medical strategy. To be the best of our knowledge, this is the first study to report the feasibility and accuracy of a risk assessment model based on the identified ten CD4^+^ MTRGs for predicting GC prognosis. These findings provide novel ideas for risk assessment in gastric cancer patients.

Despite the significant results obtained in this study, several limitations must be acknowledged. First, all data series were obtained from the GEO database in which the population distribution is mainly Caucasians, Africans, and Latinos. Therefore, caution should be taken in extrapolating the findings to patients with Asian heritage. In addition, several clinical features were not available from these databases, including severity, complications, and details of the individual treatment of each patient; therefore, we did not conduct a longitudinal analysis. Furthermore, it will be necessary to validate the ten-gene signature using external datasets. Finally, as a retrospective bioinformatic analysis study, the potential functional mechanisms of these molecular subtypes and CD4^+^ MTRGs need to be further verified in basic experimental research and clinical trials.

## Conclusion

In conclusion, three immune molecular clusters and a ten CD4^+^ MTRG signature were established for gastric cancer patients. The clusters showed significant relationships with immune status, biological processes and patient prognosis. Furthermore, a prognostic nomogram was constructed that incorporated a gene signature and independent clinical risk factors to predict the overall survival of GC patients. This nomogram might accurately identify GC patients who would benefit from immunotherapy.

## Data Availability Statement

The datasets presented in this study can be found in online repositories. The names of the repository/repositories and accession number(s) can be found in the article/**Supplementary Material**.

## Ethics Statement

Written informed consent was obtained from the individual(s) for the publication of any potentially identifiable images or data included in this article.

## Author Contributions

All authors were responsible for manuscript writing and final approval of manuscript. ZZ and T-CZ were responsible for study conception and design. Z-KN and C-GH were responsible for provision of study materials or patients. C-GH, CH, and JL were responsible for data analysis and interpretation. All authors contributed to the article and approved the submitted version.

## Funding

This study was supported by the National Natural Science Foundation of China (Grant Number: 81860433); Training Plan for Academic and Technical Young Leaders of Major Disciplines in Jiangxi Province (Grant Number: 20204BCJ23021); the Natural Science Youth Foundation of Jiangxi Province (Grant Numbers: 20192BAB215036); the Key Technology Research and Development Program of Jiangxi Province (Grant Number: 20202BBG73024); the Foundation for Fostering Young Scholar of Nanchang University (Grant Number: PY201822). Supported by National Key Clinical Discipline, Guangzhou 510655, China.

## Conflict of Interest

The authors declare that the research was conducted in the absence of any commercial or financial relationships that could be construed as a potential conflict of interest.

## Supplementary Material

Supplementary Figure 1 Workflow of this study.

Supplementary Figure 2 Kaplan-Meier analysis of 22 immunocytes.

Supplementary Figure 3 The expression of five immune checkpoint genes in three clusters **(A)** in the high- and low-risk group **(B)**.

Supplementary Figure 4 Correlation analysis between the prognostic model and immune cell infiltration.

Supplementary Figure 5 External validation of the prognostic value of the immunoscore prognostic model in GSE26899, GSE84437, GSE26901 and TCGA transcriptome data.

Supplementary Figure 6 Stratified survival analysis of the 10 DEIRG signature in different clinical features.
