# Supplementary figures and images for: Molecular Subtypes and CD4+ Memory T Cell-Based Signature Associated With Clinical Outcomes in Gastric Cancer
Source: Front Oncol. 2021 Mar 17;10:626912. doi: 10.3389/fonc.2020.626912 (PMC8011500; doi:10.3389/fonc.2020.626912)

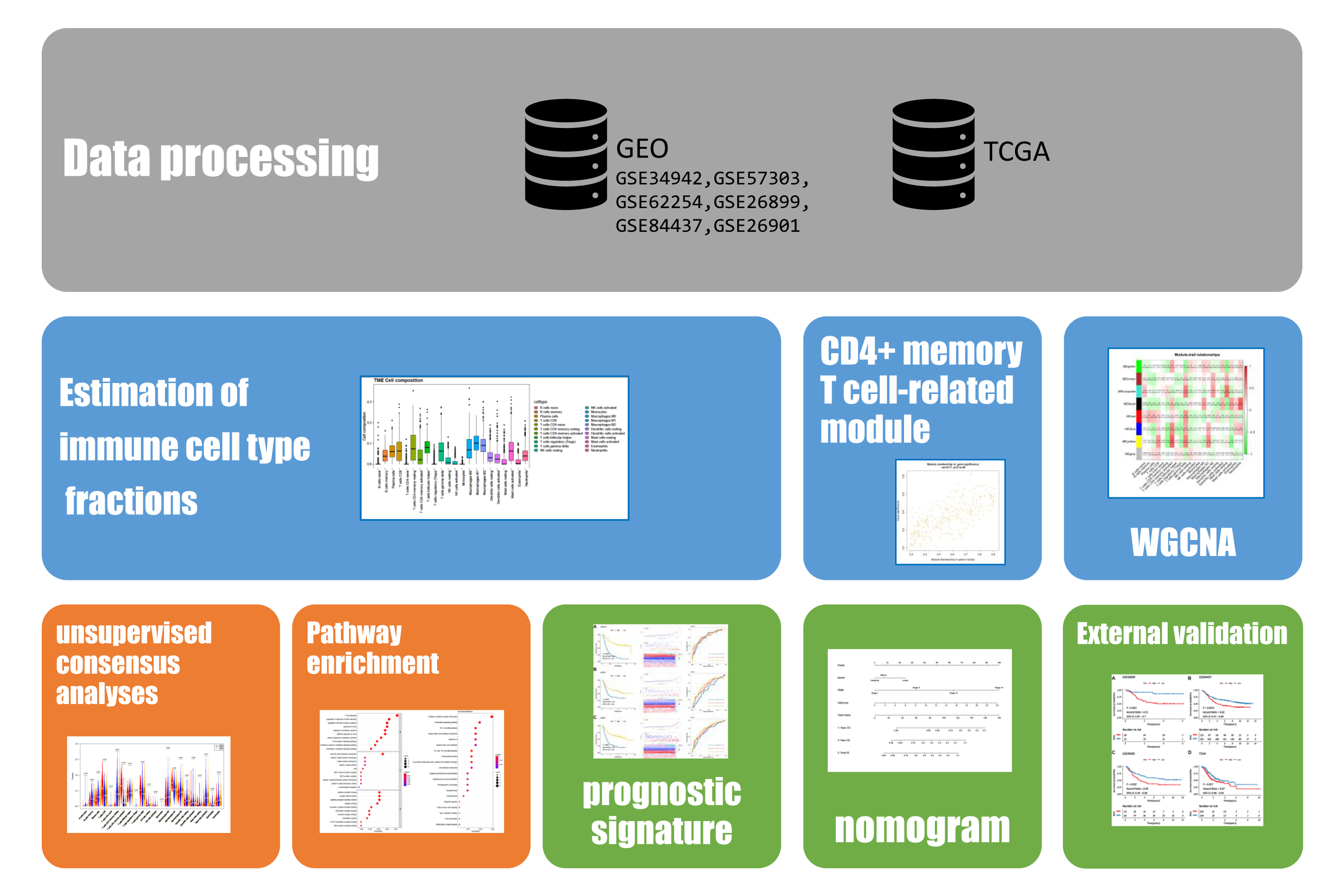

Supplement: Supplementary Figure 1 — Workflow of this study. [file Image_1.tif]

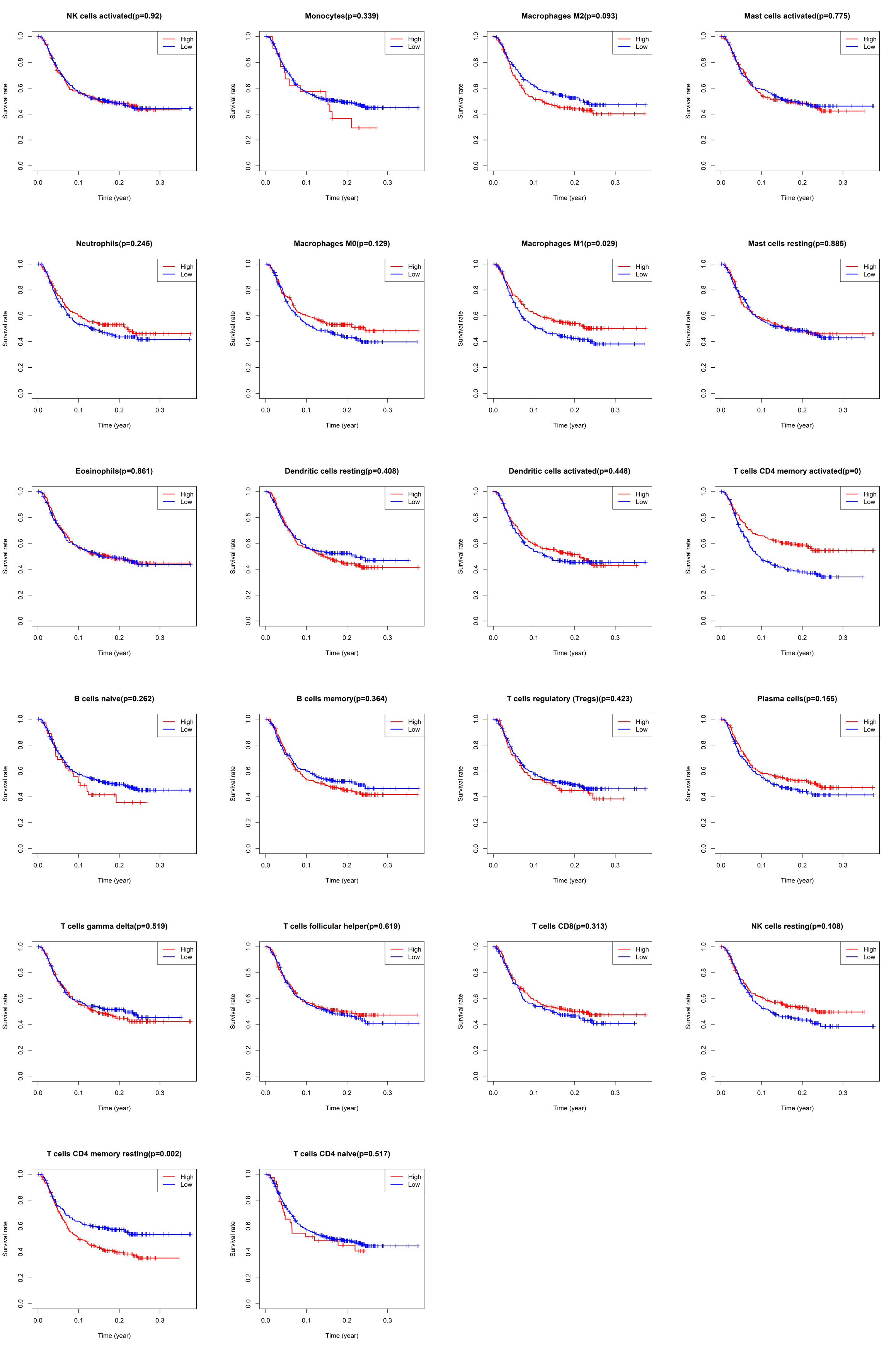

Supplement: Supplementary Figure 2 — Kaplan-Meier analysis of 22 immunocytes. [file Image_2.tif]

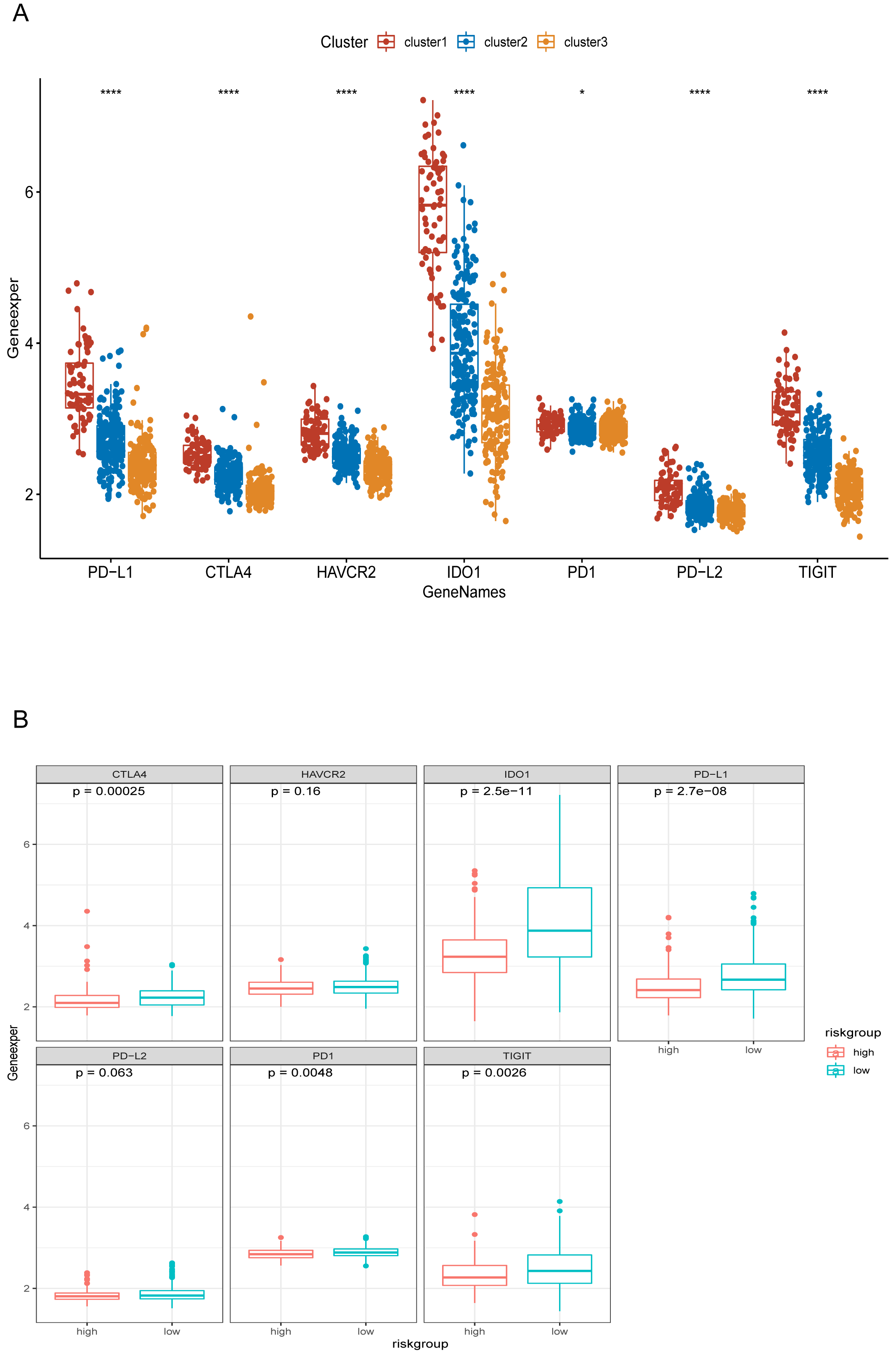

Supplement: Supplementary Figure 3 — The expression of five immune checkpoint genes in three clusters (A) in the high- and low-risk group (B). [file Image_3.tif]

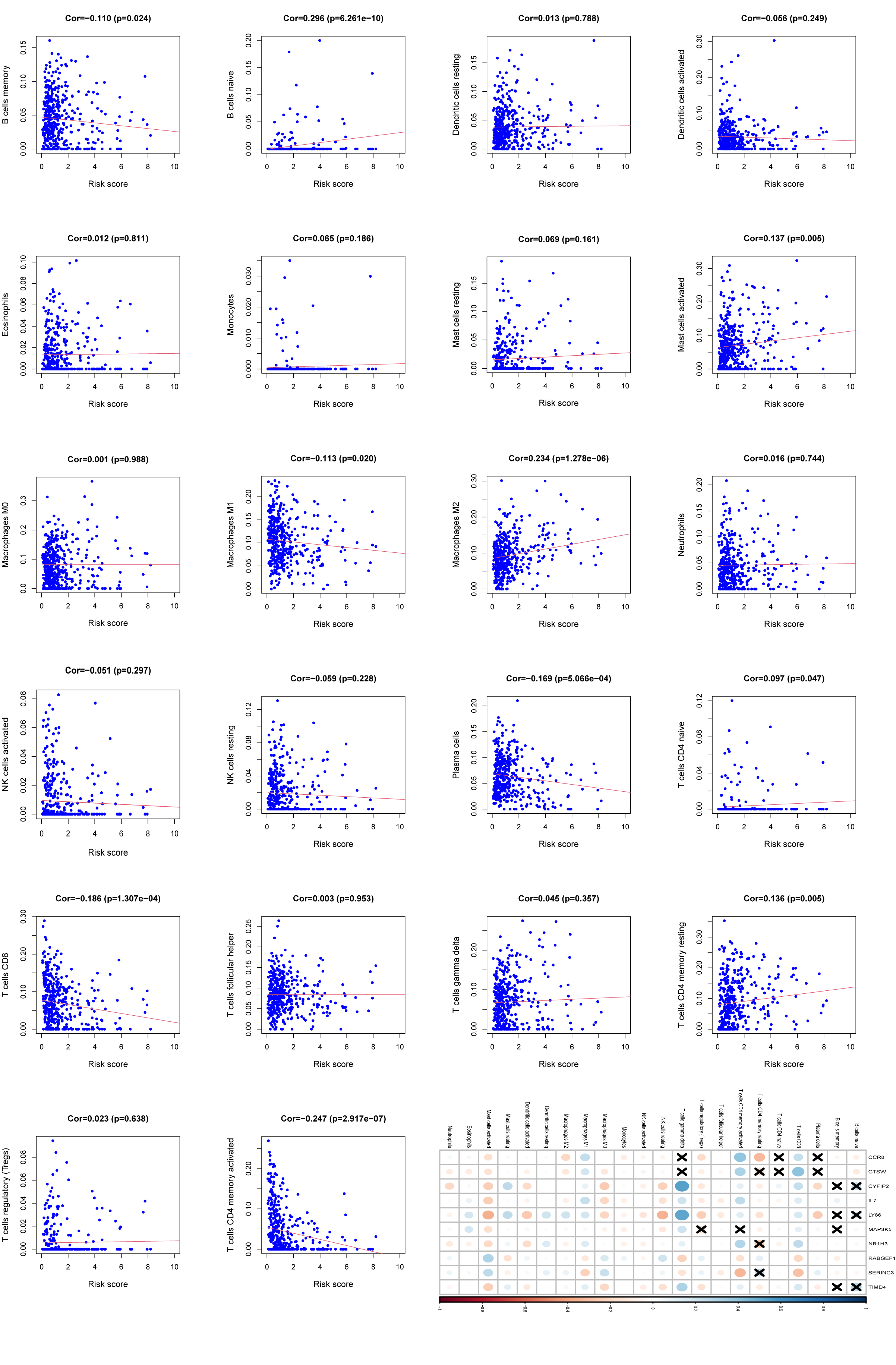

Supplement: Supplementary Figure 4 — Correlation analysis between the prognostic model and immune cell infiltration. [file Image_4.tif]

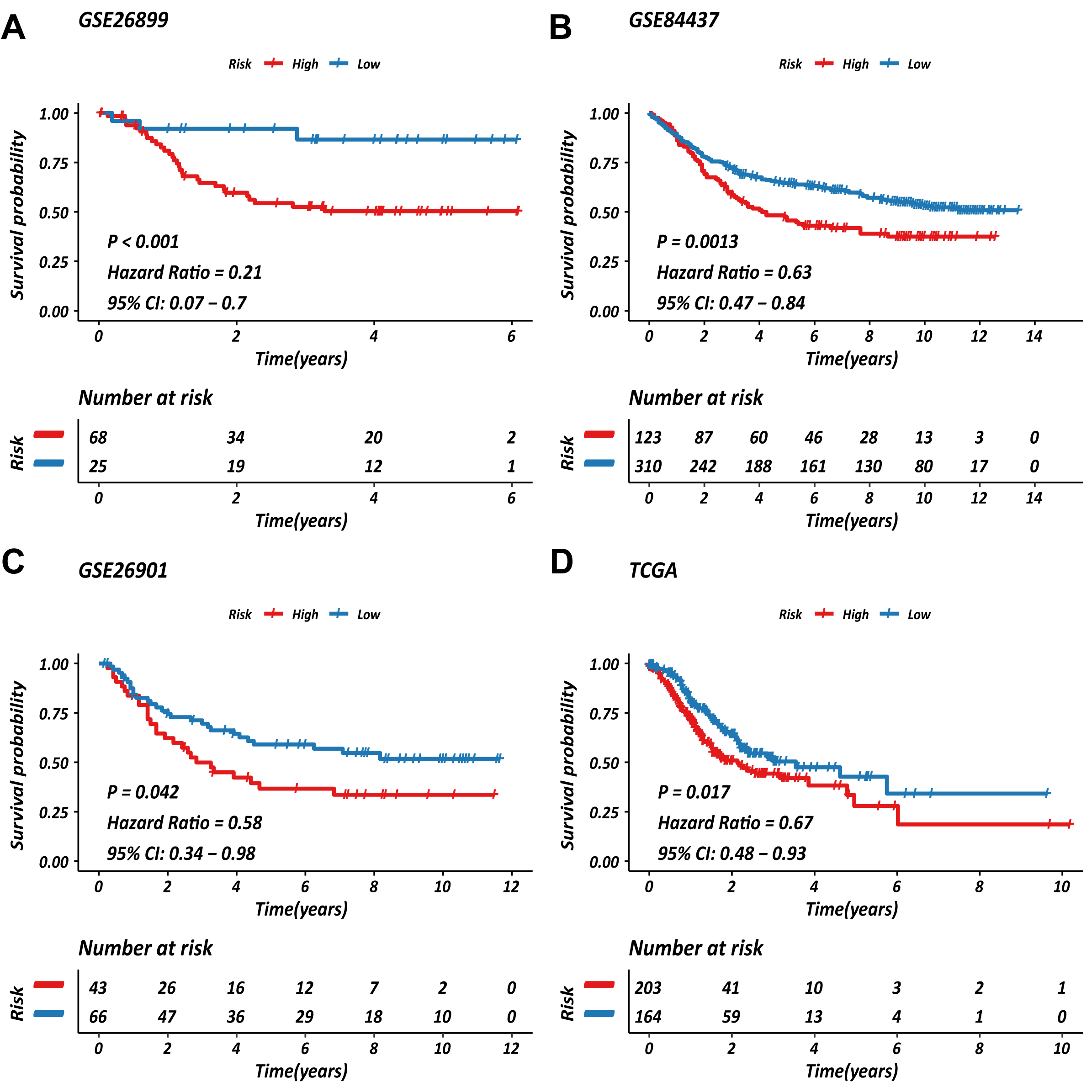

Supplement: Supplementary Figure 5 — External validation of the prognostic value of the immunoscore prognostic model in GSE26899, GSE84437, GSE26901 and TCGA transcriptome data. [file Image_5.tif]

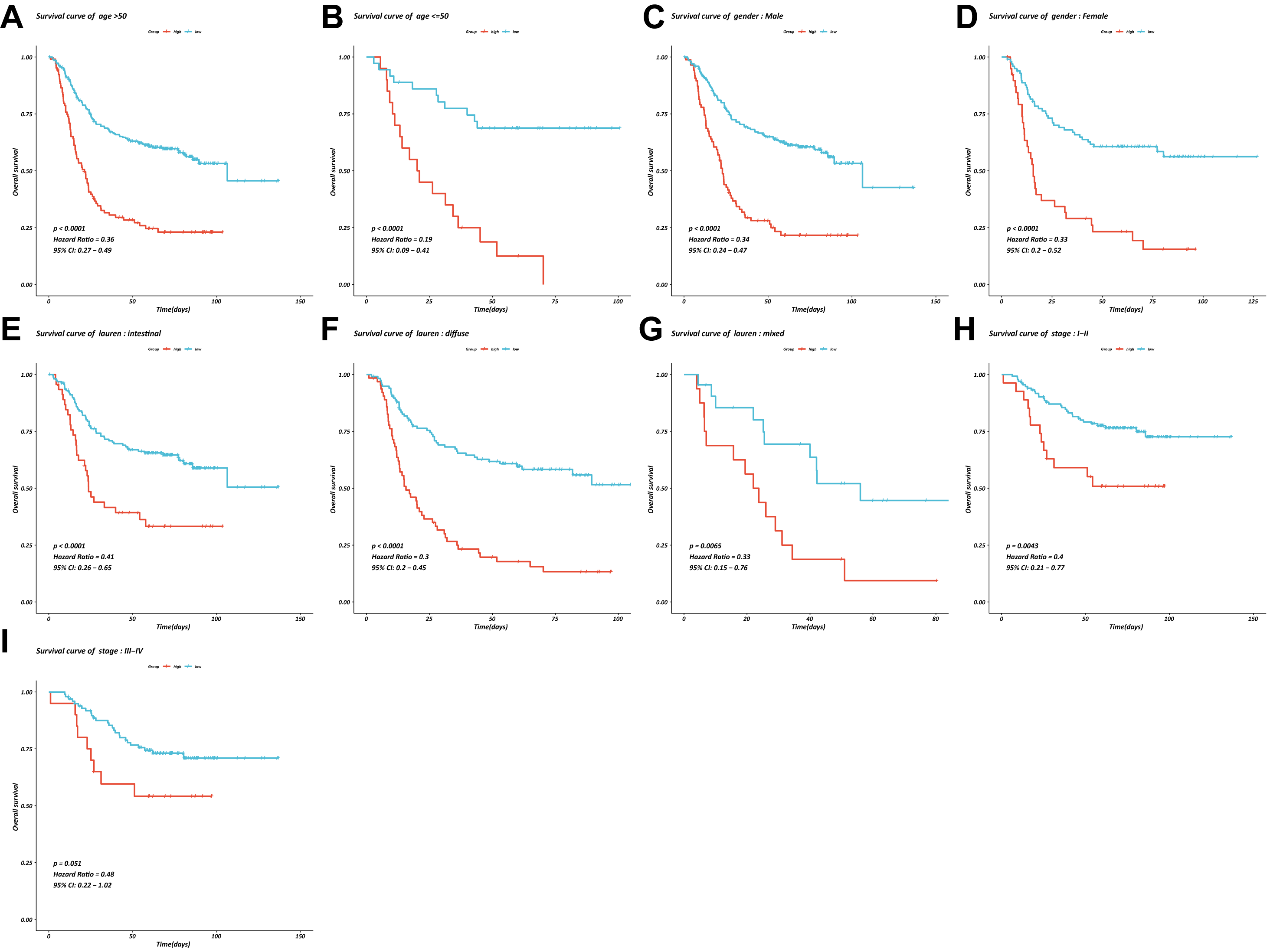

Supplement: Supplementary Figure 6 — Stratified survival analysis of the 10 DEIRG signature in different clinical features. [file Image_6.tif]
