# Supplementary material for: Molecular Subtypes and CD4+ Memory T Cell-Based Signature Associated With Clinical Outcomes in Gastric Cancer
Source: Front Oncol. 2021 Mar 17;10:626912. doi: 10.3389/fonc.2020.626912 (PMC8011500; doi:10.3389/fonc.2020.626912)
Supplement: Supplementary file 7 [file Table_1.docx]

**Supplementary Table 1 . Results of the univariate Cox regression analyses.**

| id | HR | HR.95L | HR.95H | pvalue |
| --- | --- | --- | --- | --- |
| CTSW | 0.164498162 | 0.073486667 | 0.368225236 | 1.13E-05 |
| PSMB9 | 0.56691704 | 0.42841668 | 0.750192385 | 7.16E-05 |
| PSMB8 | 0.533205386 | 0.385099028 | 0.738272399 | 0.000152087 |
| IL7 | 0.638108877 | 0.503626534 | 0.808501759 | 0.000198957 |
| TAP2 | 0.338705546 | 0.190907592 | 0.600926583 | 0.0002148 |
| TAP1 | 0.519861406 | 0.367089555 | 0.736212397 | 0.000228766 |
| CASP1 | 0.63389012 | 0.494037303 | 0.813332682 | 0.000337627 |
| CCR8 | 0.099220366 | 0.027329141 | 0.3602265 | 0.000444764 |
| SERINC3 | 3.46842075 | 1.727104383 | 6.965382416 | 0.000472266 |
| KIR2DL4 | 0.145637488 | 0.046705502 | 0.454128036 | 0.000898939 |
| IRF1 | 0.520563934 | 0.35397074 | 0.765562739 | 0.000908326 |
| UBD | 0.734827093 | 0.612470922 | 0.881626927 | 0.00091408 |
| STAT1 | 0.55466454 | 0.391422518 | 0.785986338 | 0.000919658 |
| NR1H3 | 0.459680574 | 0.289601286 | 0.729645344 | 0.000977026 |
| PSMB10 | 0.508525044 | 0.337210308 | 0.76687371 | 0.001253817 |
| IFIH1 | 0.352197433 | 0.186561084 | 0.66489232 | 0.001287182 |
| GZMB | 0.704696015 | 0.567261924 | 0.875427122 | 0.001567291 |
| SGMS1 | 0.451267985 | 0.273123441 | 0.745607163 | 0.001897743 |
| IFI35 | 0.510956295 | 0.332011303 | 0.786347732 | 0.002268022 |
| CXCL11 | 0.78244904 | 0.667596102 | 0.917061225 | 0.002454101 |
| MLF1 | 1.513030257 | 1.154998697 | 1.98204601 | 0.002647618 |
| HLA-J | 0.467170998 | 0.284086003 | 0.768248837 | 0.002710596 |
| IRF8 | 0.5776995 | 0.4009426 | 0.832380275 | 0.003234799 |
| HLA-A | 0.363836147 | 0.185319141 | 0.714317695 | 0.003310026 |
| PRKAB2 | 2.185409444 | 1.289418946 | 3.704005168 | 0.003681555 |
| PTK2B | 0.170728619 | 0.051725915 | 0.563513689 | 0.003715192 |
| PSME1 | 0.457916234 | 0.266778455 | 0.785997796 | 0.004603636 |
| ATM | 5.520512837 | 1.664689888 | 18.30735093 | 0.005219428 |
| HLA-F | 0.52677961 | 0.33535271 | 0.827477308 | 0.005404921 |
| CXCL10 | 0.750288833 | 0.612432565 | 0.919176028 | 0.005544213 |
| HLA-C | 0.424571457 | 0.231027144 | 0.780258626 | 0.005795635 |
| HLA-G | 0.483771525 | 0.28713655 | 0.815064779 | 0.006366824 |
| BIRC3 | 0.67154325 | 0.50229967 | 0.897811334 | 0.007198024 |
| GBP1 | 0.618116736 | 0.435214002 | 0.877886045 | 0.007198026 |
| CXCL9 | 0.788924469 | 0.662925121 | 0.938871975 | 0.007575439 |
| MAX | 0.283889879 | 0.112226513 | 0.718132117 | 0.007832408 |
| IDO1 | 0.776511559 | 0.64420118 | 0.935996735 | 0.007955364 |
| FKBP10 | 1.481527138 | 1.105491592 | 1.985472052 | 0.008505118 |
| KIR3DL3 | 0.217944143 | 0.069893448 | 0.679600883 | 0.008648981 |
| NCR3 | 0.077138129 | 0.01135266 | 0.524131854 | 0.008773684 |
| BST2 | 0.723333933 | 0.565437589 | 0.925322245 | 0.009947359 |
| MICB | 0.697819783 | 0.530089641 | 0.918622837 | 0.010314582 |
| LEPROTL1 | 0.490782882 | 0.281466819 | 0.855759263 | 0.012104941 |
| RABGEF1 | 2.203808894 | 1.181143766 | 4.111924203 | 0.013023325 |
| IFIT3 | 0.710074502 | 0.538042366 | 0.937111703 | 0.015570401 |
| CD247 | 0.356488855 | 0.153641327 | 0.82714922 | 0.016311595 |
| ERAP1 | 0.427685585 | 0.213372223 | 0.857257598 | 0.016661801 |
| CYFIP2 | 1.530913065 | 1.072802669 | 2.184646702 | 0.018909617 |
| LAG3 | 0.60788279 | 0.40092323 | 0.921676416 | 0.019076165 |
| TRDV3 | 0.445523546 | 0.226230954 | 0.877383165 | 0.019372467 |
| IRF9 | 0.531080565 | 0.311364681 | 0.905839949 | 0.020180829 |
| PIGT | 2.485377557 | 1.147553392 | 5.382844622 | 0.020942019 |
| HCP5 | 0.725184093 | 0.551707917 | 0.953207217 | 0.021250273 |
| HAPLN3 | 0.42560897 | 0.201328608 | 0.899737983 | 0.025313491 |
| CD244 | 0.169012945 | 0.035349544 | 0.808083297 | 0.025954944 |
| TRAFD1 | 0.284801595 | 0.092758296 | 0.874444139 | 0.0282084 |
| MAP3K5 | 0.543161126 | 0.312561639 | 0.943890651 | 0.030405117 |
| SP100 | 0.448880377 | 0.217304559 | 0.927240523 | 0.030460024 |
| CD101 | 0.214941538 | 0.053308003 | 0.86665908 | 0.030684856 |
| IL2RB | 0.640146242 | 0.424305339 | 0.965783773 | 0.033512261 |
| KLRC2 | 0.779153751 | 0.618864338 | 0.980959041 | 0.033707078 |
| HSH2D | 0.586539393 | 0.357476788 | 0.962379856 | 0.034708404 |
| IL15RA | 0.585577217 | 0.355712688 | 0.963982137 | 0.035361284 |
| HLA-B | 0.59569876 | 0.367055584 | 0.96676642 | 0.036014102 |
| NLRC5 | 0.671272563 | 0.461596452 | 0.976192194 | 0.036971758 |
| TAPBP | 0.386620404 | 0.15781093 | 0.947179877 | 0.037648318 |
| IFNG | 0.701647324 | 0.501471337 | 0.981729026 | 0.038680909 |
| HLA-DRB6 | 0.510992718 | 0.268981705 | 0.970748392 | 0.0403021 |
| IL2RG | 0.680944504 | 0.470980884 | 0.984510059 | 0.04105647 |
| CXCR3 | 0.322585805 | 0.107304732 | 0.969776445 | 0.043945335 |
| IFI27 | 0.778561956 | 0.609136475 | 0.995111513 | 0.045597301 |
| IL32 | 0.700978791 | 0.494068193 | 0.994541386 | 0.046521298 |
| PEG10 | 1.19976156 | 1.000307263 | 1.438985654 | 0.049614141 |
| CD6 | 0.138354127 | 0.018717648 | 1.022663964 | 0.052622552 |
| TSC22D3 | 2.019517376 | 0.991955248 | 4.111526647 | 0.052660336 |
| LY86 | 1.471215427 | 0.995235045 | 2.174837836 | 0.052866063 |
| JAK2 | 0.708272799 | 0.499254978 | 1.004797908 | 0.053219151 |
| CLDN3 | 0.830631259 | 0.687637747 | 1.003360113 | 0.054208922 |
| TNFRSF14 | 0.536953752 | 0.284995451 | 1.011662925 | 0.054343831 |
| ZBP1 | 0.563608334 | 0.313135936 | 1.014429576 | 0.055852159 |
| CDH17 | 0.888245821 | 0.786223294 | 1.003507075 | 0.056946144 |
| STAT2 | 0.352790001 | 0.119707734 | 1.039705461 | 0.058844331 |
| TLR3 | 0.730469618 | 0.524483357 | 1.017355184 | 0.06314575 |
| BLNK | 0.77848529 | 0.597559168 | 1.014191361 | 0.063518739 |
| IFT52 | 1.971015548 | 0.96150224 | 4.040450587 | 0.063915411 |
| IL21R | 0.55762514 | 0.299266734 | 1.039025595 | 0.065856558 |
| CD274 | 0.688087399 | 0.460875759 | 1.027314322 | 0.067522408 |
| MTCH1 | 2.374499822 | 0.937692024 | 6.012901103 | 0.068113817 |
| SECTM1 | 0.705178743 | 0.484149883 | 1.027113868 | 0.068678101 |
| GNLY | 0.780966424 | 0.59822596 | 1.019528734 | 0.069100975 |
| PLSCR1 | 0.538089426 | 0.27469107 | 1.054057672 | 0.070840458 |
| TIMD4 | 1.700209534 | 0.955515984 | 3.025289487 | 0.071043994 |
| IRF2 | 0.533713122 | 0.267138961 | 1.066297839 | 0.07537538 |
| IL12RB1 | 0.394469637 | 0.14070485 | 1.105905687 | 0.07696489 |
| NUB1 | 0.438007949 | 0.175229127 | 1.09485773 | 0.077381554 |
| IL9R | 0.181815222 | 0.027067455 | 1.221273851 | 0.079385069 |
| HLA-E | 0.569678138 | 0.300160056 | 1.08120043 | 0.085222691 |
| IFIT5 | 0.684787946 | 0.44357372 | 1.057173837 | 0.087447722 |
| TIGIT | 0.678447298 | 0.43442877 | 1.05953097 | 0.088061043 |
| CTLA4 | 0.498504385 | 0.223764165 | 1.110573813 | 0.088503044 |
| ICOS | 0.656706387 | 0.404502854 | 1.066156329 | 0.088968921 |
| PRKCB | 1.57349939 | 0.931471733 | 2.65805203 | 0.090154462 |
| CD74 | 0.632228187 | 0.371391742 | 1.076255705 | 0.091177309 |
| KLRC3 | 0.662972002 | 0.409224577 | 1.074060307 | 0.094974055 |
| KIR3DL2 | 0.321638787 | 0.083427378 | 1.240018709 | 0.099453001 |
